# Classifying diarrhea in critically ill patients through various criteria: a cohort study

**DOI:** 10.1186/s40560-025-00824-9

**Published:** 2025-09-30

**Authors:** Ryohei Yamamoto, Hajime Yamazaki, Takatoshi Koroki, Yuna Ueta, Ryo Ueno, Yosuke Yamamoto

**Affiliations:** 1 Center for Innovative Research for Communities and Clinical Excellence (CIRC2LE), Fukushima Medical University, 1 Hikarigaoka, Fukushima, Fukushima 960-1295 Japan; 2 Section of Clinical Epidemiology, Department of Community Medicine, Graduate School of Medicine, Kyoto University, Yoshida-Konoe-Cho, Sakyo-Ku, Kyoto, 606-8570 Japan; 3 Department of Intensive Care Medicine, Kameda Medical Center, 929 Higashi-Cho, Kamogawa, Chiba 296-8602 Japan; 4 Department of Nutrition Management, Ageo Central General Hospital, 1-10-10-10 Kashiwaza, Ageo-Shi, Saitama, 362-8588 Japan; 5 The Australian and New Zealand Intensive Care Research Centre, 553 St Kilda Road, Melbourne, VIC 3004 Australia; 6 Department of Healthcare Epidemiology, School of Public Health in the Graduate School of Medicine, Kyoto University, Yoshida-Honmachi, Sakyo-Ku, Kyoto, 606-8501 Japan

**Keywords:** Diarrhea, Intensive Care Units, Prevalence, Mortality

## Abstract

**Background:**

The absence of consensus criteria for diarrhea in critically ill patients poses challenges, including an uncertain prevalence and inconsistent findings regarding the impact of diarrhea on mortality. This study aimed to examine the prevalence of diarrhea, the agreement among different diagnostic criteria, and their association with mortality.

**Methods:**

A single-center cohort study was conducted among consecutive adult patients admitted to the intensive care unit (ICU) for at least three days between January 2017 and December 2018. The six diarrhea criteria evaluated were based on frequency, quantity, and consistency. These included the European Society of Intensive Care Medicine (ESICM) criteria (≥ 3 times and > 200 g/day loose or liquid stool), the World Health Organization (WHO) criteria (≥ 3 times loose or liquid stool), the Bristol Stool Chart Scale (BSCS) score of 6 or 7, and other quantity- or frequency-based definitions. Outcomes included: (1) prevalence of diarrhea according to each definition, (2) agreement between criteria, and (3) association between diarrhea and in-hospital mortality. Associations were assessed using multivariable Cox proportional hazards models, yielding hazard ratios (HRs) and 95% confidence intervals (CIs).

**Results:**

Among 700 participants, 61% were men; the median age was 71 years. The prevalence of diarrhea ranged from 9 to 39%, depending on the criteria used. The WHO and ESICM criteria showed similar prevalences (18.7% and 15.1%, respectively) and high agreement (Kappa 0.87). However, both had weak agreement with the BSCS criteria (prevalence 39.3%; Kappa 0.52 and 0.43, respectively). In univariable analyses, the presence of diarrhea was associated with in-hospital mortality, regardless of the criteria used. In multivariable analyses, only the > 400 g/day loose or liquid stool, > 200 g/day loose or liquid stool, and BSCS criteria maintained this association; the adjusted HRs (95% CI) were 1.93 (1.29‒2.90), 1.78 (1.19‒2.64), and 1.73 (1.15‒2.60), respectively.

**Conclusions:**

Diarrhea prevalence varied from 9–39% across definitions. WHO and ESICM, both frequency‑based, differed from BSCS and weight‑based criteria. Given the difficulty of accurate frequency counting in ICU patients, consistency‑ or weight‑based definitions may offer a more practical alternative for both clinical practice and research.

**Supplementary Information:**

The online version contains supplementary material available at 10.1186/s40560-025-00824-9.

## Background

Diarrhea frequently occurs as a gastrointestinal symptom in intensive care units (ICUs). It is associated with malnutrition, electrolyte imbalances, dehydration, metabolic acidosis, skin breakdown, equipment contamination, infection, and increased mortality risk [1–3]. Moreover, diarrhea has the potential to diminish patient dignity and may lead to an increased nursing workload, the need for the use of rectal tubes, and increased costs associated with testing for *Clostridioides difficile* infection (CDI). The prevalence of diarrhea in the ICU varies considerably, ranging from 10 to 78% [4, 5]. The observed discrepancy may be attributable to the use of more than 33 criteria to diagnose diarrhea in clinical practice [6], which reflects the absence of a unifying definition. These criteria differ along three diagnostic axes: (i) frequency (number of bowel movements), (ii) quantity (total stool weight), and (iii) consistency (stool form). Lack of clarity on which axis best captures clinically relevant diarrhea makes bedside recognition difficult.

An important step in addressing this problem is to develop consistent criteria for diarrhea in the ICU. The World Health Organization (WHO) defines diarrhea as having loose or liquid stools at least three times per day, focusing on the frequency and consistency of stools, and is used in many ICU studies [7–9]. However, this criterion has shortcomings in the ICU. First, where ICU patients frequently remain unconscious, the detection of diarrhea depends on the frequency of monitoring by caregivers. To make a diagnosis, a minimum of three documented observations are necessary. Second, irrespective of quantity, the presence of three or more bowel movements is indicative of diarrhea. However, the impact of diarrhea on patients can vary considerably depending on the quantity of diarrhea [10]. The European Society of Intensive Care Medicine (ESICM) Working Group therefore combined the consistency, frequency and quantity axes, defining diarrhea as passing loose or liquid stools thrice or more daily, exceeding 200–250 g per day [11].

However, there is still no clear consensus on which criteria are most relevant in the ICU setting. When defining diarrhea, it is important to assess how clinical outcomes vary, particularly in terms of its prevalence and associated mortality [12, 13]. Although previous studies have shown fair agreement between the WHO criteria and the Bristol Stool Chart Scale (BSCS), these criteria do not consider stool quantity [14].

To improve the monitoring, prevention, and management of diarrhea, this study aims to: (1) evaluate the prevalence of diarrhea in ICU settings on the basis of different criteria; (2) assess their agreement and association with mortality; and (3) generate evidence that may guide selection of the most appropriate diagnostic axis in future research and clinical care.

## Methods

### Study design and setting

This retrospective cohort study was conducted in the ICU of Kameda Medical Center. Ethical approval was obtained from the institutional review boards of both Fukushima Medical University (REC2024-030) and Kameda Medical Center (19–145). Owing to the retrospective nature of the study, these institutional review boards waived the need for informed consent from the participants. The methodology followed the Strengthening the Reporting of Observational Studies in Epidemiology (STROBE) guidelines [15].

### Study population

Between January 2017 and December 2018, we enrolled patients who stayed in the ICU for at least 3 days. During this interval the electronic medical record captured stool frequency, quantity and consistency; a system changes in February 2019 removed these fields, so later admissions could not be evaluated. Patients were excluded if they were under 18 years old, had a stoma, chronic diarrhea (such as inflammatory bowel disease), short bowel syndrome, recent gastrointestinal surgery, gastrointestinal bleeding, infectious enteritis (including CDI and cytomegalovirus enteritis), or were readmitted to the ICU.

### Outcome measurement

Our primary outcome was the prevalence of diarrhea based on the ESICM criteria, defined as a minimum of three loose or liquid bowel movements totaling more than 200 g in a day [11]. Additionally, we used five other criteria to define diarrhea as secondary outcomes: (1) three or more loose or liquid bowel movements totaling more than 400 g in a day, (2) any quantity of three or more loose or liquid bowel movements in a day (WHO criteria) [7], (3) any quantity of loose or liquid bowel movements in a day (Bristol Stool Chart Scale [BSCS] score of 6 or 7) [16], (4) more than 200 g of loose or liquid bowel movements in a day, and (5) more than 400 g of loose or liquid bowel movements in a day. The index case was defined as the first occurrence of diarrhea within the initial three days following ICU admission. This three‑day window was selected because a prior cohort study in the same ICU showed that the median time to the first episode of diarrhea was three days after admission [10]. Trained bedside nurses recorded the frequency, consistency, and quantity of stool. At this hospital, stool frequency was routinely assessed every 2 to 4 h as part of standard nursing care. Stool quantity was measured using a weight scale and documented in the electronic medical record. Daily stool output was calculated based on calendar days.

### Other data collection

Through electronic medical record review we extracted baseline characteristics obtained within the first 24 h of ICU admission, including age, sex, patient category (medical, surgical, or trauma), admission diagnosis, presence of sepsis [13], the Charlson Comorbidity Index (CCI) [17], the Acute Physiology and Chronic Health Evaluation II (APACHE II) score [18], and the Sequential Organ Failure Assessment (SOFA) score [19]. For ICU days 1 to 3 we recorded daily treatments that could contribute to diarrhea, namely proton pump inhibitors, enteral nutrition, antibiotics and laxatives, and we noted whether enteral nutrition or laxatives had commenced before the first diarrhea episode. We also collected data on ICU, in‑hospital, 28‑day and 90‑day mortality, as well as the length of ICU and hospital stays and ventilator‑free days within the first 90 days [20].

### Statistical analysis

Patient characteristics were presented as the median and interquartile range (IQR). The prevalence of diarrhea was the proportion of patients who developed diarrhea between days 1 and 3. We used the percentage agreements and the kappa statistic to assess agreements across the six diarrhea criteria. Interpretation of the kappa values was categorized as follows: 0 to 0.20 indicates no agreement, 0.21 to 0.39 indicates minimal agreement, 0.40 to 0.59 indicates weak agreement, 0.60 to 0.79 indicates moderate agreement, 0.80 to 0.90 indicates strong agreement, and above 0.90 signifies almost perfect agreement [21].

To assess the relationship between diarrhea and in-hospital mortality, we applied Cox proportional hazards models, estimating hazard ratios (HRs) along with 95% confidence intervals (CIs). The proportional-hazard assumption was verified using the Schoenfeld residual method. To conduct time-to-event analyses, data from patients who were lost to follow-up were censored. The multivariable analysis incorporated adjustments for several factors: age, sex, CCI, SOFA score, enteral nutrition, and antibiotic use. The selection of these covariates was predetermined, on the basis of their clinical relevance [4, 5, 14, 22]. When the percentage of missing values exceeded 5%, we planned to address the missing data by implementing multiple imputations via the chained equations method (MICE) [23].

Our study included three sensitivity analyses: First, we added norepinephrine use as a covariate to the adjustment model. Second, we conducted an analysis limited to patients without risk of urine and stool contamination (anuria, urinary catheter, nephrostomy catheter, or cystostomy catheter). Finally, we used 90-day mortality as the outcome instead of in-hospital mortality. As a post-hoc analysis, we repeated the primary multivariable Cox model, replacing the SOFA score with APACHE II score and adding mechanical ventilation use as a covariate to approximate opioid exposure. Given the retrospective design, the entire eligible cohort was analyzed without an a priori sample‑size calculation. All the statistical analyses used R software, version 4.4.2, courtesy of the R Foundation for Statistical Computing, Vienna, Austria (https://www.R-project.org/).

## Results

### Patient characteristics

During the study period, 1584 patients were admitted to the ICU, and 872 patients met the inclusion criteria. Of these, 172 patients were excluded, resulting in a final cohort of 700 patients for analysis (Supplementary Fig. 1, Additional File 1). The median (IQR) age was 71 (63–79) years, the SOFA score was 7 (5–10), and 451 (64%) patients were mechanically ventilated at baseline on study day 1 (Table 1). Among patients who received enteral nutrition, 96% were given Mei Balance HP 1.0^®^, delivered continuously via nasogastric tube. None of the patients received probiotics or synbiotics during the observation window. Diarrhea classified by the ESICM criteria was present in 106 (15%) patients. Patients with diarrhea had increased severity, sepsis diagnosis, noradrenaline use, and enteral nutrition use. Among the majority of patients (691/700; 99%), a urinary or renal catheter was present, or they were receiving maintenance dialysis or were anuric.

**Table 1:** Baseline characteristics of the study patients

Characteristic	Overall N = 700	No diarrhea N = 594	Diarrhea N = 106
Age, Median (IQR)	71 (63–79)	71 (63–78)	70 (63–81)
Male, n (%)	449 (64)	382 (64)	67 (63)
CCI, Median (IQR)	1 (0–3)	1 (0–3)	2 (1–3)
APACHE II score*, Median (IQR)	16 (12–24)	15 (11–23)	23 (18–29)
SOFA score*, Median (IQR)	7 (5–10)	7 (4–10)	9 (7–12)
Serum albumin, g/dl, Median (IQR)	3.0 (2.5–3.3)	3.0 (2.6–3.4)	2.6 (2.1–3.0)
Type of patients, n (%)			
Medical	338 (48)	252 (42)	86 (81)
Surgical	328 (47)	309 (52)	19 (18)
Trauma	34 (4.9)	33 (5.6)	1 (0.9)
Admission diagnosis, n (%)			
Cardiovascular	250 (36)	241 (41)	9 (8.5)
Sepsis	220 (31)	149 (25)	71 (67)
Respiratory	55 (7.9)	52 (8.8)	3 (2.8)
Trauma	34 (4.9)	33 (5.6)	1 (0.9)
Neurological	33 (4.7)	30 (5.1)	3 (2.8)
Metabolic	28 (4.0)	22 (3.7)	6 (5.7)
Genitourinary	11 (1.6)	9 (1.5)	2 (1.9)
Gastro-intestinal	9 (1.3)	8 (1.3)	1 (0.9)
Hematologic	6 (0.9)	2 (0.3)	4 (3.8)
Other	54 (7.7)	48 (8.1)	6 (5.7)
Infection site (sepsis cases), n (%)			
Respiratory	94 (43)	61 (41)	33 (46)
Genitourinary	31 (14)	18 (12)	13 (18)
Gastrointestinal	24 (11)	17 (11)	7 (9.9)
Skin or Musculoskeletal	20 (9.1)	17 (11)	3 (4.2)
Unknown	15 (6.8)	12 (8.1)	3 (4.2)
Other	36 (16)	24 (16)	12 (17)
Renal replacement therapy, n (%)	112 (16)	89 (15)	23 (22)
Mechanical ventilation, n (%)	451 (64)	397 (67)	54 (51)
Noradrenaline, n (%)	267 (38)	197 (33)	70 (66)
Vasopressin, n (%)	52 (7.4)	33 (5.6)	19 (18)
Enteral nutrition, n (%)	278 (40)	214 (36)	64 (60)
Antibiotics, n (%)	617 (88)	519 (87)	98 (92)
laxative, n (%)	363 (52)	329 (55)	34 (32)
Proton pump inhibitors, n (%)	582 (83)	489 (82)	93 (88)

*IQR* Interquartile range, *CCI* Charlson Comorbidity Index, *APACHE II* Acute Physiology and Chronic Health Evaluation II, *SOFA* Sequential Organ Failure Assessment

Diarrhea was defined according to ESICM criteria, as having at least three loose or liquid bowel movements totaling more than 200 g in a day

^*^Nine missing data

There were no missing values in other variables

### Prevalence of diarrhea on the basis of different criteria

**Fig. 1:**
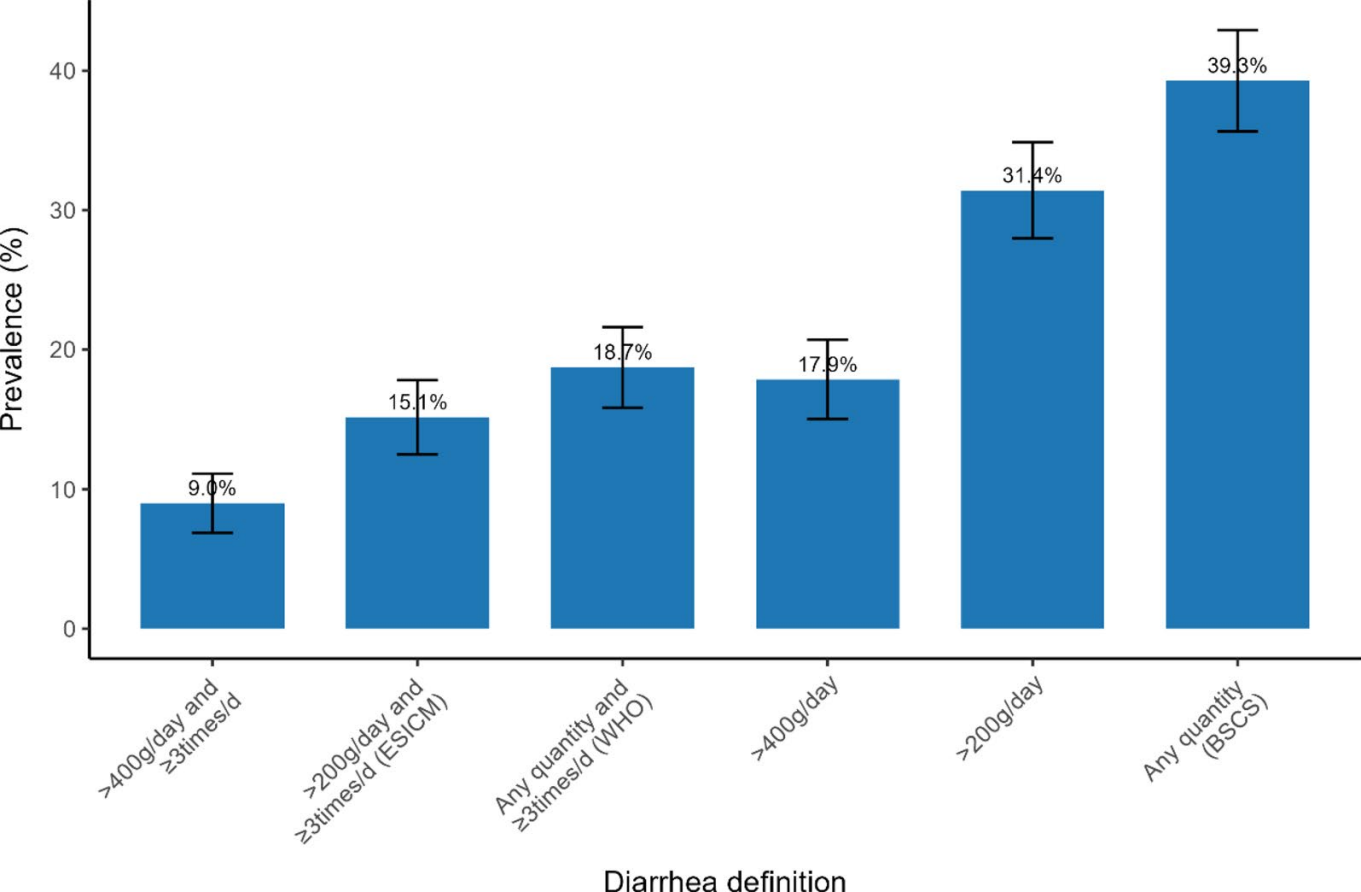
Prevalence based on various diarrhea criteria

When the six diarrhea criteria were applied to this cohort, the prevalence of diarrhea ranged between 9 and 39%, as summarized in Fig. 1. The highest prevalence was observed with the BSCS criteria (any quantity of loose or liquid bowel movement per day), at 39.3% (95% CI 35.7% to 42.9%). The WHO criteria (any quantity and ≥ 3 times loose or liquid bowel movements per day) had a prevalence of 18.7% (95% CI 15.8 to 21.6%), whereas the ESICM criteria (> 200 g/day and ≥ 3 times loose or liquid bowel movements per day) had a prevalence of 15.1% (95% CI 12.5 to 17.8%).

### Agreement of diarrhea criteria

The highest percentage agreement was observed between the WHO criteria and the ESICM criteria at 96% (Table 2). Moderate percentage agreement was also observed between the BSCS criteria and the WHO criteria at 79%, and between the BSCS criteria and the ESICM criteria at 76%. Among the kappa coefficients, the highest value (0.87) was found between the WHO criteria and the ESICM criteria, indicating strong agreement. Weak agreement was observed between the BSCS criteria and the WHO criteria with a kappa of 0.52, and between the BSCS criteria and the ESICM criteria, with a kappa of 0.43. The agreement between the BSCS criteria and > 200 g/day was 0.83.

**Table 2:** Agreements and kappa values for various diarrhea criteria

Criteria	> 400 g/day and ≥ 3times/d	> 400 g/day	> 200 g/day and ≥ 3times/d (ESICM)	> 200 g/day	Any quantity and ≥ 3times/d (WHO)	Any quantity (BSCS)
> 400 g/day and ≥ 3times/d		0.63	0.71	0.35	0.6	0.27
> 400 g/day	91		0.53	0.64	0.5	0.5
> 200 g/day and ≥ 3times/d (ESICM)	94	87		0.56	0.87	0.43
> 200 g/day	78	86	84		0.56	0.83
Any quantity and ≥ 3times/d (WHO)	90	85	96	83		0.52
Any quantity (BSCS)	70	79	76	92	79	

Upper right is the kappa values and lower left is the percentage agreements

The kappa values were categorized as follows: 0 to 0.20 indicates no agreement, 0.21 to 0.39 minimal, 0.40 to 0.59 weak, 0.60 to 0.79 moderate, 0.80 to 0.90 strong, and above 0.90 signifies almost perfect agreement [18]

### Mortality and length of stay

**Fig. 2:**
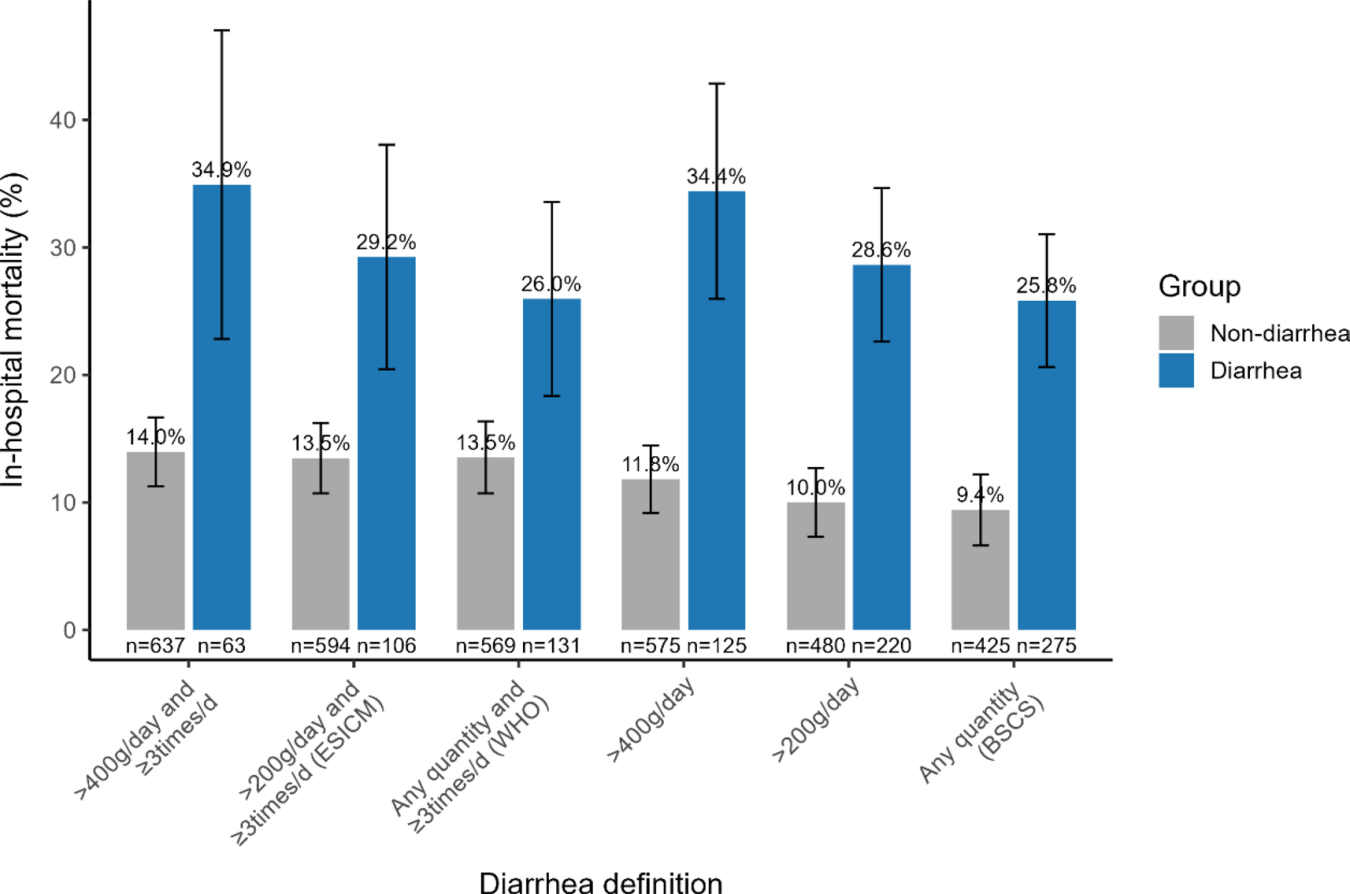
In-hospital mortality based on various diarrhea criteria

Patients with diarrhea classified according to the ESICM criteria had a longer hospital stay, with a median of 34 days (IQR 18–54), and an in-hospital mortality of 29% (Table 3). When the six diarrhea criteria were applied to this cohort, in-hospital mortality ranged from 26 to 35% in patients with diarrhea (Fig. 2). For diarrhea defined by the WHO criteria, the in-hospital mortality was 26%. For diarrhea defined by the BSCS criteria, the in-hospital mortality was 26% (Supplementary Table 1, Additional File 1).

**Table 3:** Length of stay and mortality

Characteristic	Overall N = 700	No diarrhea N = 594	Diarrhea N = 106
ICU length of stay, Median (IQR)	4.0 (3.0–7.0)	4.0 (3.0–7.0)	5.0 (4.0–8.0)
Hospital length of stay, Median (IQR)	21 (14–45)	21 (13–43)	34 (18–54)
ICU mortality, n (%)	45 (6.4)	30 (5.1)	15 (14)
In-hospital mortality, n (%)	111 (16)	80 (13)	31 (29)
28-day mortality, n (%)	76 (11)	53 (9.0)	23 (22)
Lost to follow-up	5	5	0
90-day mortality, n (%)	112 (17)	79 (14)	33 (34)
Lost to follow-up	41	32	9

*IQR* Interquartile range

Diarrhea was defined according to ESICM criteria, as having at least three loose or liquid bowel movements totaling more than 200 g in a day

### Associations between various criteria and mortality

**Fig. 3:**
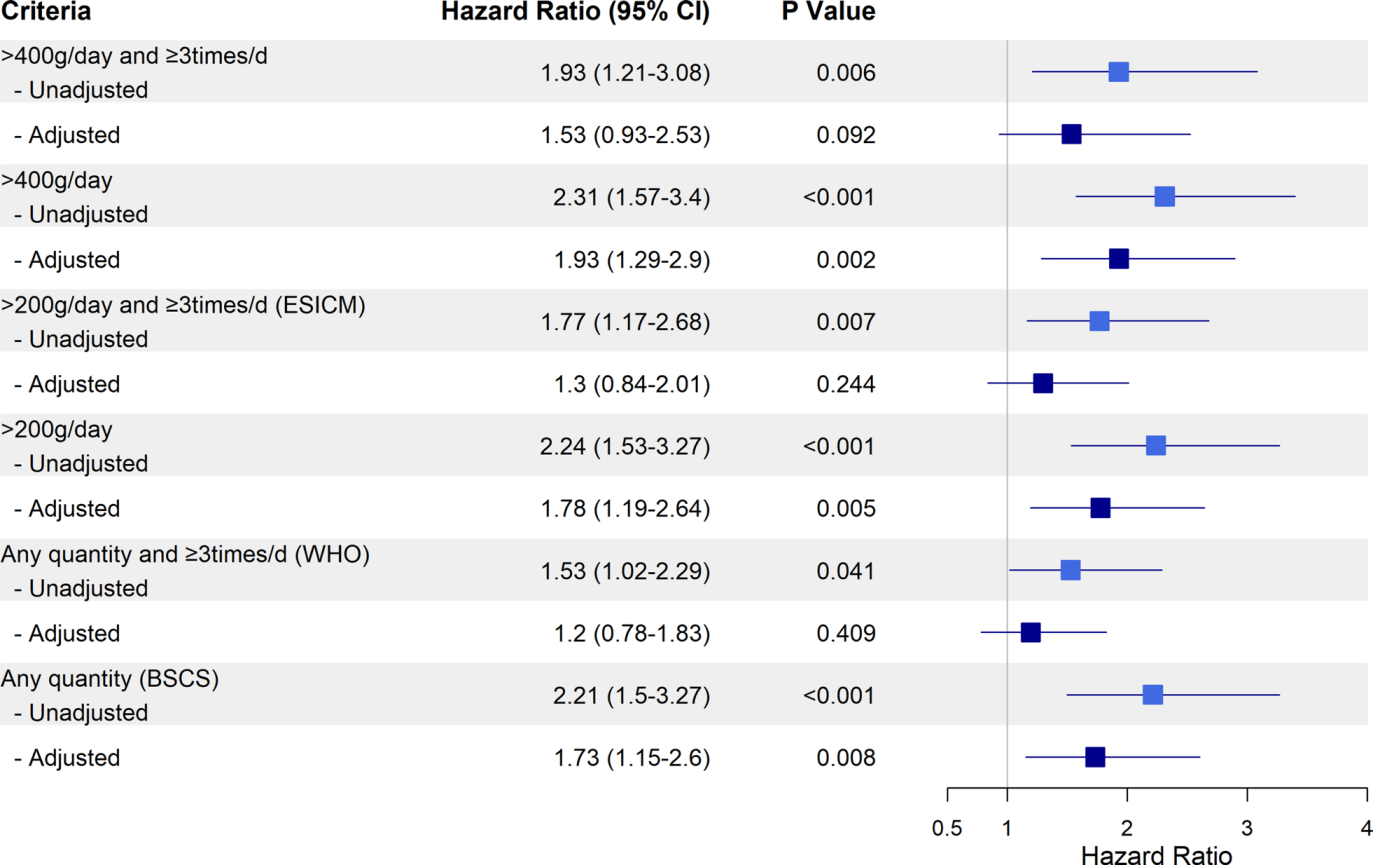
Associations between diarrhea criteria and in-hospital mortality. Adjusted for age, sex, CCI, SOFA score, enteral nutrition, and antibiotic use

Our dataset was nearly complete, with less than 2% missing data, so imputation was not needed. According to the unadjusted analyses, all diarrhea criteria were associated with increased in-hospital mortality (Fig. 3). For the ESICM criteria, the unadjusted analysis indicated an increased risk of in-hospital mortality (HR, 1.77; 95% CI 1.17–2.68; P = 0.007). According to the WHO criteria, the unadjusted HR was 1.53 (95% CI 1.02–2.29; P = 0.041). For the BSCS criteria, the unadjusted analysis revealed an increased mortality risk (HR, 2.21; 95% CI 1.50–3.27; P < 0.001). After adjusting for covariates, only the criteria of more than 400 g/day (HR, 1.93; 95% CI 1.29–2.90; P = 0.002), more than 200 g/day (HR, 1.78; 95% CI 1.19–2.64; P = 0.005), and the BSCS criteria (HR, 1.73; 95% CI 1.15–2.60; P = 0.008) remained associated with increased risk. Sensitivity analyses revealed that both the unadjusted and adjusted results were similar to those of the primary analysis (Supplementary Figs. 2 and 3, Additional File 1). The diarrhea criteria were associated with 90-day mortality, except for the WHO and ESCIM criteria (Supplementary Fig. 4, Additional File 1). A post‑hoc model that substituted APACHE II for SOFA and added mechanical ventilation as a covariate produced only minor attenuation of effect sizes (Supplementary Fig. 5, Additional File 1).

## Discussion

### Summary of key findings

The study revealed that the prevalence of diarrhea ranged from 9 to 39%, depending on the criteria used. The WHO and ESICM criteria had similar prevalence and showed strong agreement, whereas both showed weak agreement with the BSCS criteria. The BSCS criteria, in turn, demonstrated strong agreement with the > 200 g day criterion. To our knowledge, this is the first study to evaluate quantity‑based thresholds (> 200 g day and > 400 g day) in an ICU cohort. In the univariable analyses, all criteria were associated with mortality. However, in the multivariable analyses, only the > 400 g day, > 200 g day, and BSCS criteria retained this association, suggesting that these weight‑ and consistency‑based definitions may be better predictors of mortality risk than frequency‑based criteria.

### Context in association with the published literature

The findings of this study are consistent with and contribute to the literature on the variability of diarrhea criteria and their implications. The present study demonstrated that the application of multiple criteria to the same cohort resulted in different prevalences of diarrhea. In particular, the BSCS criteria identified a higher prevalence of diarrhea compared to the WHO and ESICM criteria, capturing a broader spectrum of diarrhea cases. The largest prior study reported different incidences during ICU stays using WHO criteria, and BSCS, supporting the present findings [14]. Although our study reported lower prevalences of diarrhea compared to previous studies, this discrepancy may be attributed to differences in the observation periods. A number of previous studies did not specify the timing of diarrhea onset, suggesting that the timing of observation may also influence the prevalence of diarrhea [8].

When evaluating the agreement of the criteria for diarrhea, it was found that some criteria agreed, whereas others were not. Some studies suggest the use of the ESICM criteria, which are frequency- and quantity-based, or the WHO criteria [3, 11, 24]. Our results demonstrated that the ESICM criteria were largely consistent with the WHO criteria. Moreover, the two sets of criteria yield comparable estimates of the association with mortality, which may render either criterion acceptable for use. In contrast, the ESICM and WHO criteria for diarrhea showed weak agreement with the BSCS criteria, a finding that aligns with those of previous studies [14]. The primary distinction between these criteria pertains to the assessment of diarrhea frequency. In the ICU, stool frequency could be an unreliable measure because it depends on how often staff inspect diapers, and it is virtually impossible to record when a rectal tube is in place. As a result, frequency-based definitions, such as the WHO criterion, appear to undercount diarrhea. In our analysis, these definitions detected fewer cases and had a weaker association with mortality, while weight- and consistency-based criteria retained a clear prognostic signal. These findings suggest that clinically significant episodes are likely to be missed when frequency is used and support the adoption of weight- or consistency-based definitions for routine surveillance and future research. A further consideration is that stool frequency may not accurately reflect disease severity, whereas stool quantity is more likely to do so. Repeated passes of small quantities are typically clinically not important, whereas a single large‑quantity evacuation can lead to dehydration or electrolyte disturbances and may therefore confer a greater risk of adverse outcomes [10]. Quantifying stool output, which may better capture the physiological burden of diarrhea, could aid in identifying clinically significant cases. These observations therefore suggest that quantity‑ or consistency‑based definitions merit consideration for routine surveillance and future investigations.

Although the ESICM criteria identified diarrhea cases with the highest absolute in-hospital mortality, the adjusted hazard ratio for mortality was higher when using the BSCS definition. This apparent discrepancy may be explained by the composition of the comparator (non-diarrhea) groups. The BSCS criteria classify a larger proportion of low-risk patients as non-diarrhea, thereby increasing the contrast with the diarrhea group and yielding a higher hazard ratio. In contrast, the more stringent ESICM criteria result in a comparator group that includes patients with moderate severity, leading to a smaller difference in hazard despite higher absolute mortality among those classified as diarrhea.

The BSCS and quantity-defined criteria (> 200 g/day and > 400 g/day) had relatively greater associations with mortality risk than the other criteria did, suggesting that these criteria may be more reliable in identifying high-risk patients. Previous studies have reported conflicting results regarding the association between diarrhea and increased risk of death [8, 9]. One reason may be the influence of variations in the criteria. In our study, all diarrhea criteria consistently tended to increase the risk of death, but the strength of the association varied by criterion.

Diarrhea independently associated with mortality, yet this link is likely prognostic rather than causal. Two complementary mechanisms may explain the association. First, diarrhea can signal gastrointestinal organ dysfunction. Patients who develop diarrhea typically show higher illness‑severity scores than those who do not [8, 25], and the relationship in our cohort persisted after adjustment for the SOFA score, implying that diarrhea captures prognostic information not reflected in standard organ severity indices. A possible reason why diarrhea is associated with death is that patients with diarrhea have more severe disease, which may reflect changes in intestinal perfusion and the gut microbiota during critical illness [25, 26]. Second, diarrhea may represent dysbiosis driven by critical illness, broad‑spectrum antimicrobials and enteral nutrition, a state that increases vulnerability to infection, multiorgan failure and death [26, 27]. Although our dataset cannot directly examine these pathways, their biological plausibility highlights the need for future studies to determine whether diarrhea is a modifiable target or simply an indicator of underlying disease severity.

Because exposure classification was fixed at day 3 to reduce immortal-time bias [28, 29], the reported associations quantify the prognostic value of early-onset diarrhea rather than diarrhea occurring later in the ICU stay. Some patients without diarrhea at the landmark may have developed diarrhea after day 3. If the risk associated with late-onset diarrhea is similar to, or greater than, the risk associated with early-onset diarrhea, excluding these later episodes would tend to dilute the observed associations. Conversely, if late-onset diarrhea arises predominantly among lower-risk survivors, its prognostic strength may differ from that observed in the early window. For these reasons the present findings should not be over-generalized to late-onset episodes, and future work should employ designs that specifically capture them, such as time-varying exposure models or sequential landmarks with adequate power [30].

### Implications for clinicians and future research

Determining which criteria should be used for diarrhea is an important issue. In clinical practice, the BSCS may have advantages if it broadly identifies diarrhea cases. In the present study, we evaluated a criterion that considers quantity, showing that the BSCS and > 200 g/day criteria were similar in terms of prevalence and association with mortality. This finding supports the use of the BSCS criterion even in situations where quantities cannot be measured. On the other hand, the WHO criteria and ESICM criteria might miss cases of diarrhea by considering frequency as a criterion. Methods that do not use frequency as a criterion (e.g., BSCS) may be simpler and easier to use than methods that require accurate frequency counts over 24 h. These results can inform clinical practice and research by encouraging the adoption of standardized criteria for identifying and managing diarrhea. Future work should confirm these findings in larger cohorts, including disease-specific groups such as patients with sepsis, and should assess intermediate outcomes such as nutritional adequacy, interruptions of enteral feeding, skin integrity, and infection events to capture the full clinical impact of diarrhea and guide targeted interventions.

### Limitations

This study has several limitations. First, the generalizability of a single-center study is limited, because differing patient backgrounds and disease severity may influence diarrhea prevalence. Second, for technical reasons we extracted data by calendar day, which may have underestimated stool frequency, because episodes that began late at night were truncated at midnight and any further evacuations were counted on the following day. Third, by restricting diarrhea ascertainment to the first three ICU days, late-onset episodes were likely missed, potentially attenuating measured associations with outcomes. Fourth, although the formulation of enteral nutrition was identified, pre-ICU nutritional intake, infused volume, and daily caloric intake were not recorded. Fifth, pathophysiology and antibiotic exposures likely differ across clinical subgroups such as sepsis and postoperative patients; biomarker data, for example interleukin-6, and detailed antibiotic stratification were unavailable, so unmeasured covariates may have influenced both the occurrence of diarrhea and its prognostic significance. Sixth, the reliability and validity of the BSCS have not been formally established in ICU settings. Finally, the study may have been under-powered to detect associations between the WHO or ESICM criteria and mortality.

## Conclusions

The prevalence of diarrhea ranged from 9 to 39% depending on the criteria used. While the WHO and ESICM criteria gave similar results, they differed from those based on the BSCS criteria. Definitions based on stool consistency or weight appeared to perform better than frequency-based criteria and may be more appropriate for use in the ICU. In both clinical and research settings, the choice of criteria should take into account factors such as prevalence, agreement with other measures, and their association with mortality.

## Supplementary Information

Additional file 1.

## Data Availability

The data that support the findings of this study are available from the corresponding author upon reasonable request.

## Abbreviations

AKI: Acute Kidney Injury
APACHE II: Acute Physiology and Chronic Health Evaluation II
BSCS: Bristol Stool Chart Scale
CCI: Charlson Comorbidity Index
CDI: Clostridioides difficile infection
CI: Confidence interval
ESICM: European Society of Intensive Care Medicine
HR: Hazard ratio
ICU: Intensive care unit
IQR: Interquartile range
MICE: Multivariable Imputation by Chained Equations
SOFA: Sequential Organ Failure Assessment
STROBE: Strengthening the Reporting of Observational Studies in Epidemiology
WHO: World Health Organization
