# Supplementary material for: Classifying diarrhea in critically ill patients through various criteria: a cohort study
Source: J Intensive Care. 2025 Sep 30;13:55. doi: 10.1186/s40560-025-00824-9 (PMC12487300; doi:10.1186/s40560-025-00824-9)
Supplement: Supplementary file 1 — Additional file 1. [file 40560_2025_824_MOESM1_ESM.docx]

**Supplemental materials**

**Classifying Diarrhea in Critically Ill Patients Through Various Criteria: A cohort Study**

Ryohei Yamamoto^1, 2, 3^, Hajime Yamazaki^2^, Takatoshi Koroki^3^, Yuna Ueta^4^, Ryo Ueno^5^, Yosuke Yamamoto^5*^

^1^ Center for Innovative Research for Communities and Clinical Excellence (CIRC2LE), Fukushima Medical University, 1 Hikarigaoka, Fukushima, Fukushima 960-1295, Japan

^2^ Section of Clinical Epidemiology, Department of Community Medicine, Graduate School of Medicine, Kyoto University, Yoshida-konoe-cho, Sakyo-ku, Kyoto 606-8570, Japan

^3^ Department of Intensive Care Medicine, Kameda Medical Center, 929 Higashi-cho, Kamogawa, Chiba, 296-8602, Japan

^4^ Department of nutrition management, Ageo Central General Hospital, 1-10-10-10 Kashiwaza, Ageo-shi, Saitama 362-8588, Japan

^5^ The Australian and New Zealand Intensive Care Research Centre, 553 St Kilda Road, Melbourne VIC 3004, Australia

^6^ Department of Healthcare Epidemiology, School of Public Health in the Graduate School of Medicine, Kyoto University, Yoshida-honmachi, Sakyo-ku, Kyoto 606-8501 Japan

**Corresponding Author:**

Ryohei Yamamoto

Center for Innovative Research for Communities and Clinical Excellence (CIRC2LE), Fukushima Medical University, Fukushima, Japan.

1 Hikarigaoka, Fukushima, Fukushima 960-1295, Japan

Tel: +81- 024-547-1467

Fax: +81- 024-547-1468

E-mail: [ryoheiyamamoto11@gmail.com](mailto:ryoheiyamamoto11@gmail.com)

Supplementary Figure. 1. Flow diagram of patient selection.
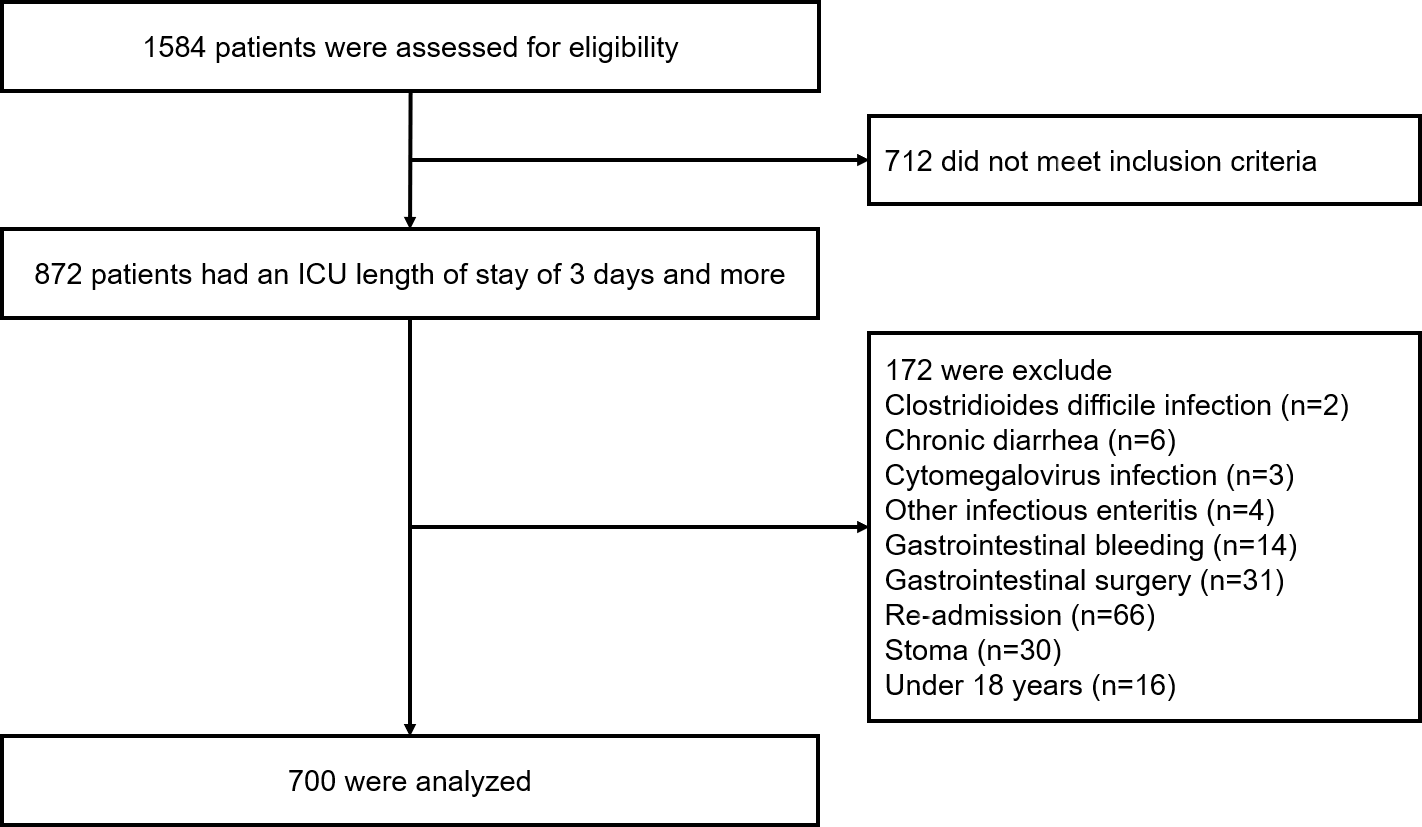


Supplementary Figure 2. Association Between Diarrhea Criteria and In-hospital Mortality (Noradrenaline added to covariates)


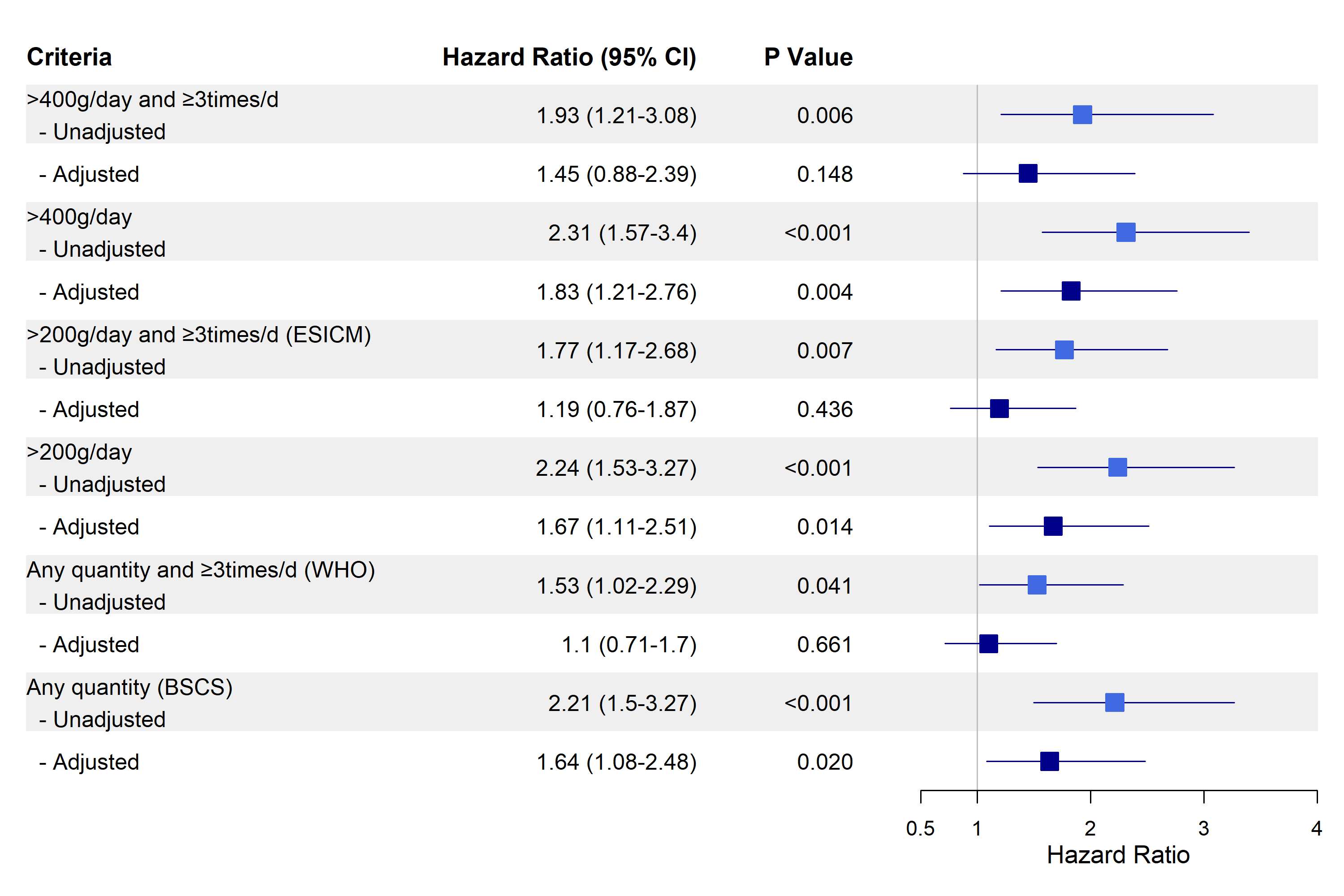


Supplementary Figure2 Legend: Adjusted for age, sex, CCI, SOFA score, enteral nutrition, antibiotic use, and noradrenaline use.

Supplementary Figure 3. Association Between Diarrhea Criteria and In-hospital Mortality without Risk of Urine and Stool Contamination
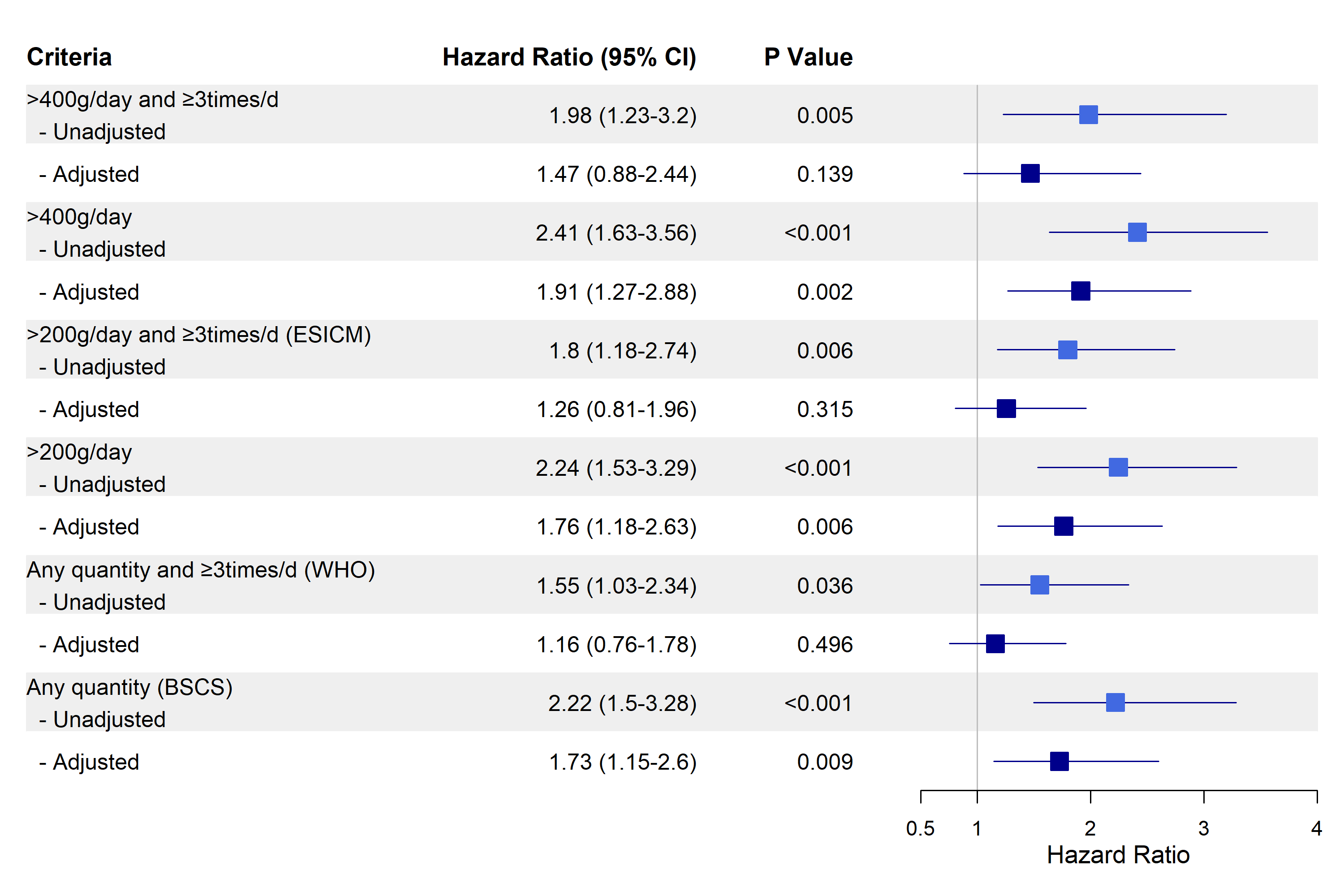


Supplementary Figure 3 legend: Adjusted for age, sex, CCI, SOFA score, enteral nutrition, and antibiotic use.

Supplementary Figure 4. Association Between Diarrhea Criteria and 90-day Mortality 
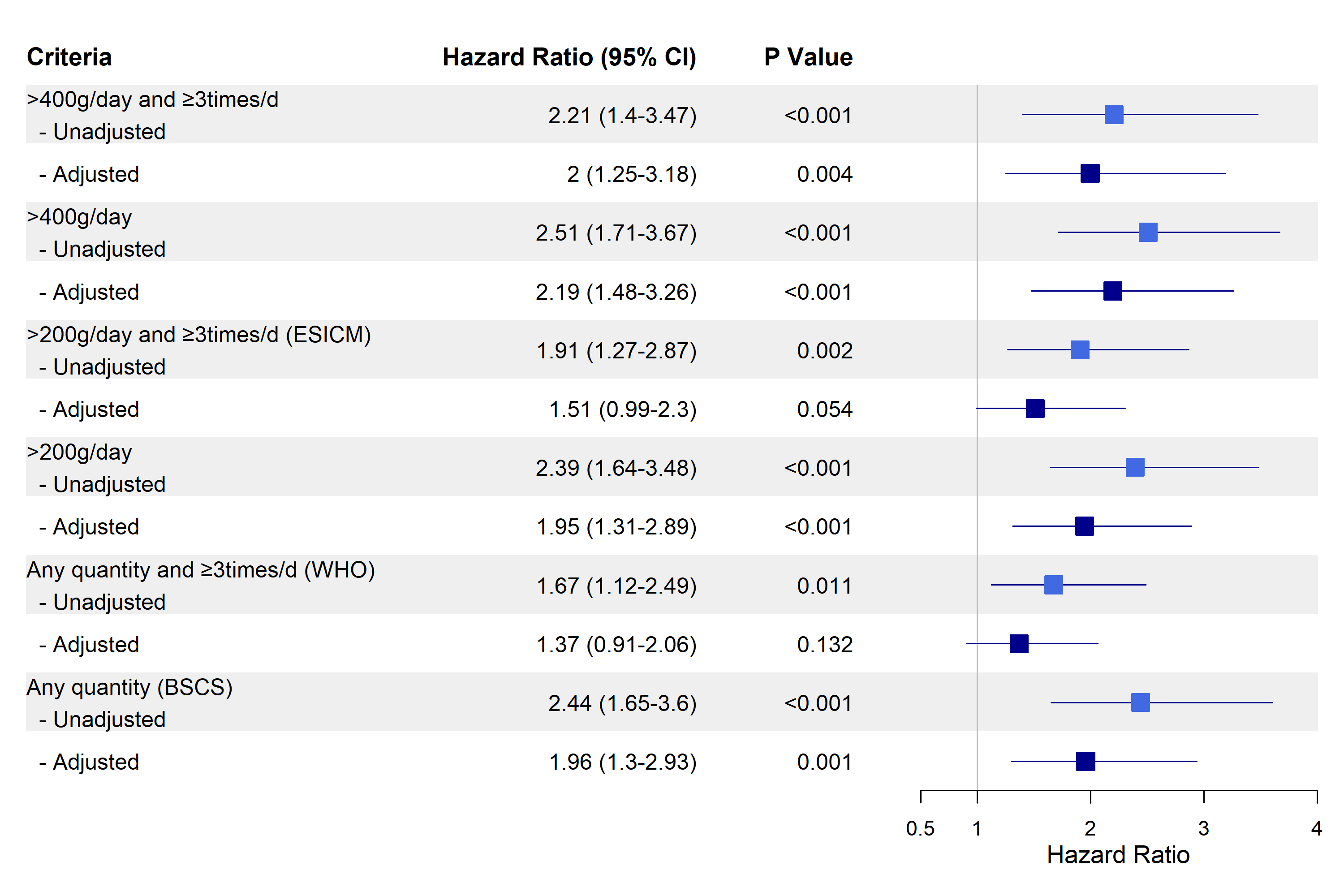


Supplementary Figure 4 legend: Adjusted for age, sex, CCI, SOFA score, enteral nutrition, and antibiotic use.

Supplementary Figure 5. Association Between Diarrhea Criteria and In-hospital Mortality (APACHE II and mechanical ventilation added as covariates)


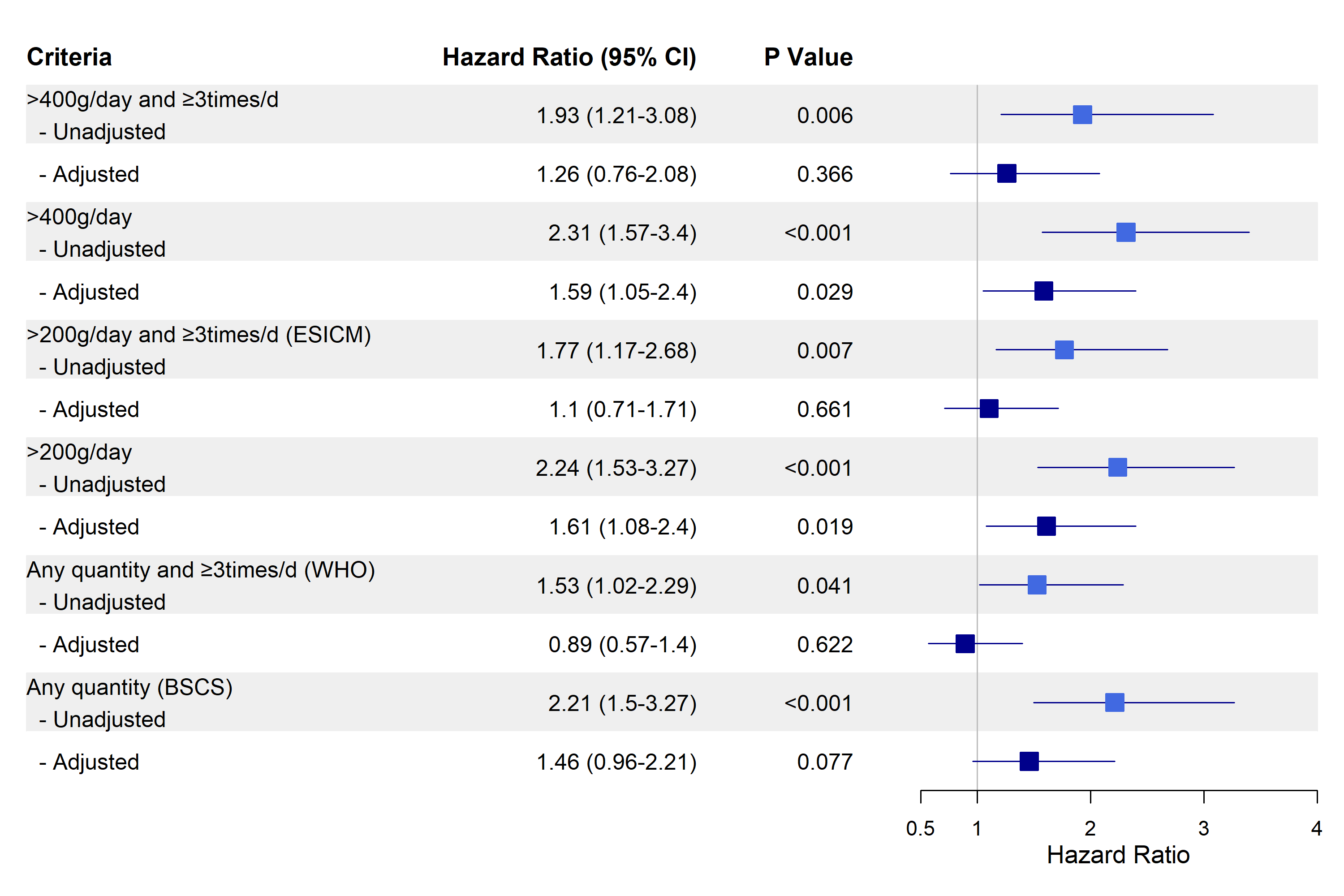


Supplementary Figure 5 legend: Adjusted for age, sex, CCI, APACHE 2 score, enteral nutrition, antibiotic use, mechanical ventilation use.

Supplementary Table 1. Length of Stay and Mortality Based on Various Diarrhea Criteria

|  | >400g/day and   ≥3times/d | >400g/day | >200g/day and   ≥3times/d (ESICM) | >200g/day | Any quantity and   ≥3times/d (WHO) | Any quantity   (BSCS) |
| --- | --- | --- | --- | --- | --- | --- |
| **Characteristic** | **N = 63** | **N = 125** | **N = 106** | **N = 220** | **N = 131** | **N = 275** |
| Cumulative Days^*^, Median [IQR]) | 1 [1-2] | 1 [1-2] | 1 [1-2] | 2 [1-3] | 2 [1-3] | 2 [1-4] |
| ICU length of stay, Median (IQR) | 6.0 (4.0 – 8.0) | 5.0 (4.0 – 8.0) | 5.0 (4.0 – 8.0) | 5.0 (4.0 – 8.0) | 5.0 (4.0 – 7.0) | 5.0 (4.0 – 8.0) |
| Hospital length of stay, Median (IQR) | 40 (19 – 55) | 30 (17 – 54) | 34 (18 – 54) | 30 (16 – 54) | 33 (18 – 54) | 27 (15 – 52) |
| ICU mortality, n (%) | 10 (16) | 17 (14) | 15 (14) | 28 (13) | 16 (12) | 32 (12) |
| In-hospital mortality, n (%) | 22 (35) | 43 (34) | 31 (29) | 63 (29) | 34 (26) | 71 (26) |
| 28-day mortality, n (%) | 16 (25) | 31 (25) | 23 (22) | 46 (21) | 24 (18) | 52 (19) |
| Lost to follow-up |  | 2 |  | 3 |  | 3 |
| 90-day mortality, n (%) | 24 (40) | 44 (37) | 33 (34) | 64 (31) | 36 (30) | 73 (29) |
| Lost to follow-up | 3 | 6 | 9 | 16 | 11 | 21 |
| IQR: Interquartile range.  ^*^Cumulative diarrhea days were calculated over ICU days 1 to 7. | | | | | | |
